# Mantle Cell Lymphoma of the Thyroid: A Rare Cause of Life‐Threatening Airway Compromise

**DOI:** 10.1002/kjm2.70048

**Published:** 2025-05-15

**Authors:** Kanokwan Kesornpatumanant, Nantawan Numkiatsakul, Narittee Sukswai, Thiti Snabboon

**Affiliations:** ^1^ Department of Medicine Phra Nakhon Si Ayutthaya Hospital Phra Nakhon Si Ayutthaya Thailand; ^2^ Department of Medicine, Faculty of Medicine Chulalongkorn University Bangkok Thailand; ^3^ Department of Pathology, Faculty of Medicine Chulalongkorn University Bangkok Thailand; ^4^ Excellence Center in Diabetes, Hormone and Metabolism, King Chulalongkorn Memorial Hospital, Thai Red Cross Society Bangkok Thailand

1

A 67‐year‐old Thai woman presented with a rapidly enlarging neck mass over two months. She was afebrile but demonstrated stridor and accessory muscle use during breathing. The thyroid mass was markedly enlarged, hard, and non‐tender, with bilateral cervical lymphadenopathy. Emergency intubation was performed to secure the airway due to respiratory distress. CT scan revealed an infiltrative thyroid mass extending into the mediastinum, causing significant tracheal compression (Figure 1), along with multiple lymphadenopathies in the cervical, intrathoracic, and pelvic regions. Lymph node biopsy confirmed stage IIIE mantle cell lymphoma (MCL) (Figure 1B). Laboratory tests revealed slightly elevated LDH, normal thyroid function, and negative thyroid antibodies. Initial treatment with dexamethasone followed by R‐CHOP chemotherapy led to a significant reduction in the neck mass within a week, allowing successful extubation (Figure 1C). There was no evidence of recurrence or metastasis at 8 months after 6 cycles of the R‐CHOP regimen.

**FIGURE 1:**
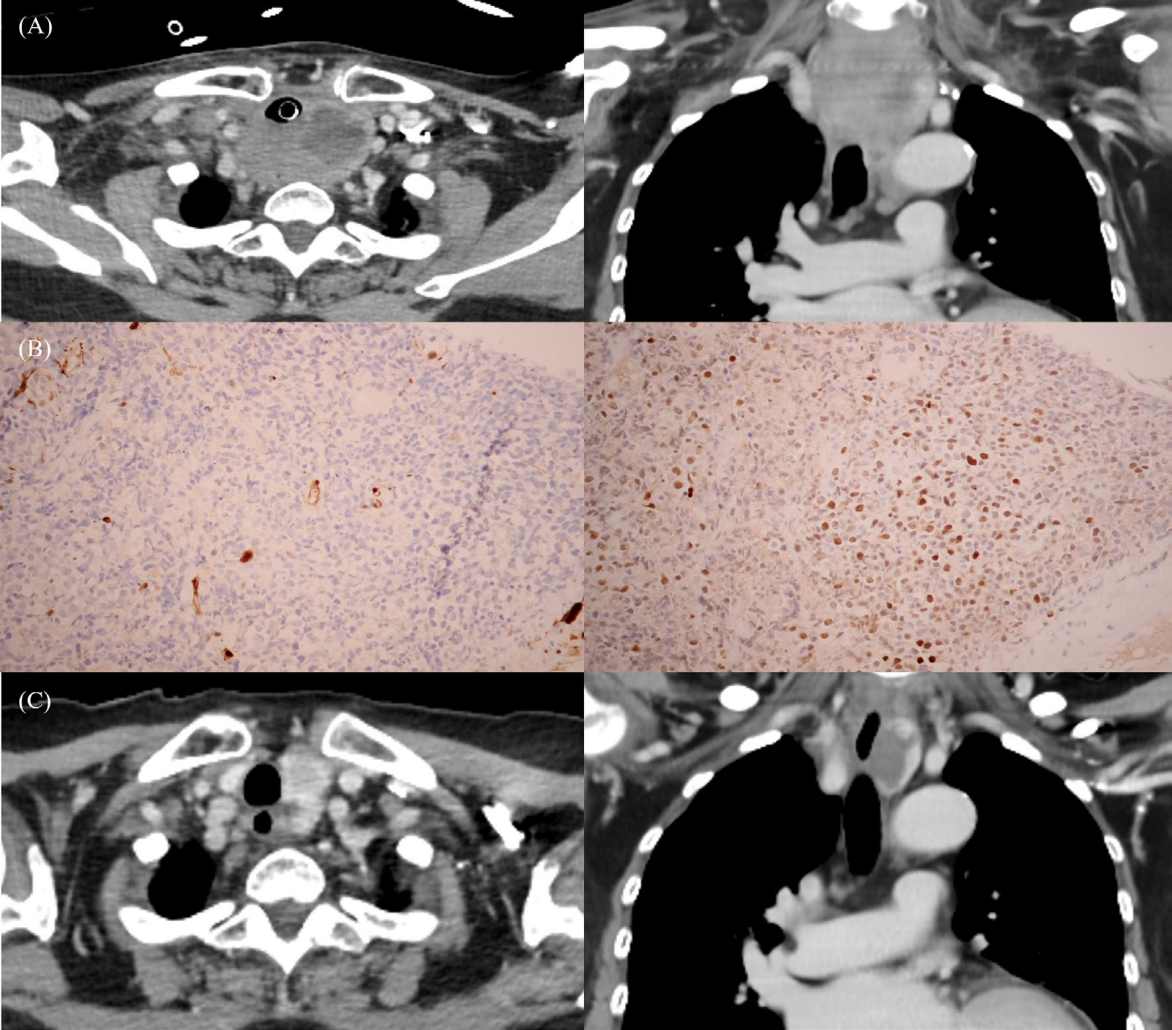
(A) CT scan of the neck (axial and sagittal views) showed a large thyroid mass with internal necrosis, extending into the superior mediastinum and significantly narrowing the tracheal lumen. The presence of an endotracheal tube was noted. (B) Immunohistochemistry showed positive SOX 11 expression (right) and negative cyclin D1 expression (left). (C) CT scan of the neck after 3 months of treatment showed significant resolution of the infiltrative thyroid mass, now appearing as a lobulated hypodense lesion with enhancing mural nodules in the inferior pole of the left lobe, causing a mild rightward trachea shift.

A rapidly enlarging thyroid mass presents diagnostic challenges due to the risk of life‐threatening airway obstruction and the potential for aggressive malignancies, such as anaplastic thyroid carcinoma (ATC) and thyroid lymphoma. Other possible etiologies include thyroid abscess, cystic degeneration, and Riedel's thyroiditis. Prompt diagnosis is critical, involving imaging studies, such as USG or CT, and tissue diagnosis through FNA or core‐needle biopsy for accurate management.

Primary thyroid lymphoma (PTL) is a rare malignancy, representing 0.4%–5% of thyroid cancers and 2% of extranodal lymphomas, with an incidence of 0.5 per 100,000 individuals [1]. It predominantly affects women in their sixth decade, with Hashimoto thyroiditis increasing the risk up to 80‐fold. Over 70%–90% of cases present with a rapidly enlarging goiter, and 10%–25% experience significant airway compromise. Thyroid function is usually normal, but hypothyroidism occurs in 10% due to Hashimoto's thyroiditis, and thyrotoxicosis is rare. Ultrasonographic features are nonspecific, overlapping with other thyroid malignancies. FNA is useful for initial assessment, but core needle or surgical biopsy is often required for definitive diagnosis. Prognosis depends on histology, tumor extent, disease stage, serum LDH levels, and performance status. Treatment typically involves rituximab with chemotherapy, often supplemented by radiotherapy, while surgery is reserved for diagnosis or palliative relief of compressive symptoms.

Diffuse large B‐cell lymphoma (DLBCL) is the most common subtype of PTL, accounting for 50%–70% of cases and exhibiting a more aggressive course compared to MALT or follicular lymphomas [2]. MCL, a rare subtype of non‐Hodgkin lymphoma (NHL), represents less than 10% of cases and is even rarer as PTL [3]. Known for its aggressive behavior, MCL often presents at advanced stages, with a median survival of 3–5 years. MCL can be classified into two distinct forms: nodal MCL, which accounts for 80%–90% of cases and typically expresses SOX‐11, and a non‐nodal form with a more indolent course [4]. In advanced‐stage MCL, as illustrated in our case, the R‐CHOP chemotherapy regimen, commonly used for DLBCL, is effective. Obstructive symptoms often improve rapidly after treatment initiation, mainly due to the steroid component [5]. This case highlights the importance of accurate diagnosis and timely treatment in preventing airway compromise caused by mass compression, often avoiding the need for tracheostomy.

## Conflicts of Interest

The authors declare no conflicts of interest.

## Data Availability

Data sharing not applicable to this article as no datasets were generated or analysed during the current study.
